# Tat-malate dehydrogenase fusion protein protects neurons from oxidative and ischemic damage by reduction of reactive oxygen species and modulation of glutathione redox system

**DOI:** 10.1038/s41598-023-32812-0

**Published:** 2023-04-06

**Authors:** Hyun Jung Kwon, Kyu Ri Hahn, Min Soo Kang, Jung Hoon Choi, Seung Myung Moon, Yeo Sung Yoon, In Koo Hwang, Dae Won Kim

**Affiliations:** 1 Department of Biochemistry and Molecular Biology, Research Institute of Oral Sciences, College of Dentistry, Gangneung-Wonju National University, Gangneung, 25457 South Korea; 2 Department of Biomedical Sciences, and Research Institute for Bioscience and Biotechnology, Hallym University, Chuncheon, 24252 South Korea; 3 Department of Anatomy and Cell Biology, College of Veterinary Medicine, and Research Institute for Veterinary Science, Seoul National University, Seoul, 08826 South Korea; 4 Department of Anatomy, College of Veterinary Medicine and Institute of Veterinary Science, Kangwon National University, Chuncheon, 24341 South Korea; 5 Department of Neurosurgery, Kangnam Sacred Heart Hospital, College of Medicine, Hallym University, Seoul, 07441 South Korea; 6 Research Institute for Complementary & Alternative Medicine, Hallym University, Chuncheon, 24253 South Korea

**Keywords:** Neuroscience, Cell death in the nervous system, Diseases of the nervous system

## Abstract

Malate dehydrogenase (MDH) plays an important role in the conversion of malate to oxaloacetate during the tricarboxylic acid cycle. In this study, we examined the role of cytoplasmic MDH (MDH1) in hydrogen peroxide (H_2_O_2_)-induced oxidative stress in HT22 cells and ischemia-induced neuronal damage in the gerbil hippocampus. The Tat-MDH1 fusion protein was constructed to enable the delivery of MDH1 into the intracellular space and penetration of the blood–brain barrier. Tat-MDH1, but not MDH1 control protein, showed significant cellular delivery in HT22 cells in a concentration- and time-dependent manner and gradual intracellular degradation in HT22 cells. Treatment with 4 μM Tat-MDH1 significantly ameliorated 200 μM H_2_O_2_-induced cell death, DNA fragmentation, and reactive oxygen species formation in HT22 cells. Transient increases in MDH1 immunoreactivity were detected in the hippocampal CA1 region 6–12 h after ischemia, but MDH1 activity significantly decreased 2 days after ischemia. Supplementation of Tat-MDH1 immediately after ischemia alleviated ischemia-induced hyperlocomotion and neuronal damage 1 and 4 days after ischemia. In addition, treatment with Tat-MDH1 significantly ameliorated the increases in hydroperoxides, lipid peroxidation, and reactive oxygen species 2 days after ischemia. Tat-MDH1 treatment maintained the redox status of the glutathione system in the hippocampus 2 days after ischemia. These results suggest that Tat-MDH1 exerts neuroprotective effects by reducing oxidative stress and maintaining glutathione redox system in the hippocampus.

## Introduction

Transient cerebral ischemia is one of the most prevalent cardiovascular diseases and is ranked among the top five leading causes of death in the United States^1^. Interruption of blood vessels and reperfusion have detrimental effects on various cell organelles, including mitochondria, and energy production^2–4^. Glucose is the main energy source for brain metabolism during glycolysis and mitochondrial respiration. Under normal conditions, reactive oxygen species (ROS) are generated in the mitochondria through oxidative phosphorylation, whereas ischemia/reperfusion causes an enormous increase in ROS production^5,6^. Therefore, enzymes related to the tricarboxylic acid (TCA) cycle are inevitably exposed to oxidative damage after ischemia/reperfusion. In the gerbils, transient forebrain ischemia causes a delay in the mitochondrial redox ratio in the hippocampal CA1 region and dentate gyrus in the early period, but this delay persists in the CA1 region^7^.

ROS induced by ischemia/reperfusion cause degenerative protein modification of amino acids containing cysteine (Cys) residues, which are the catalytic sites of various enzymes^8,9^. Oxidative stress causes the sequential oxidation of Cys to sulfenic acid, sulfinic acid, and sulfonic acid, whereas thioredoxin reverses Cys oxidation to the reduced state to prevent hyperoxidation and irreversible redox regulation^10,11^. However, the mitochondrial redox ratio is persistently reduced in the hippocampal CA1 region after ischemia^7^.

Malate dehydrogenase (MDH, EC 1.1.1.37), a key enzyme in the TCA cycle, catalyzes the conversion of malate to oxaloacetate by the reduction of nicotinamide adenine dinucleotide (NAD) + hydrogen (H) (NADH) from NAD + . Cytoplasmic and mitochondrial MDH (MDH1 and MDH2, respectively) have been introduced and synthesized in the cytoplasm. MDH1 remains in the cytoplasm and is expressed in the brain, heart, and skeletal muscle^12^. MDH1 has been related to various neurological disorders including sporadic Creutzfeldt-Jakob disease^13^, nicotine toxicity^14^, hypoxia^15,16^, and schizophrenia^17–19^. MDH1 is increased in the serum after myocardial infarction in humans^20^, whereas it is decreased in the hippocampus 24 h after hypoxic damage^15^.

Recently, MDH1 was identified as a major target for irreversible oxidation (trioxidized at Cys137) in aged brains, and its activity was found to be significantly lower in aged brains^21^. In a previous study, we demonstrated that thioredoxin immunoreactivity was decreased in the gerbil hippocampal CA1 region 6 h after ischemia, whereas hyperoxidized peroxiredoxins and glyceraldehyde-3-phosphate dehydrogenase levels were significantly increased in the gerbil hippocampal CA1 region 12–24 h after ischemia^22^, suggesting irreversible oxidative damage in proteins containing Cys residues. However, no studies have examined the changes and effects of MDH1 after transient forebrain ischemia in the gerbils. In the present study, we uncovered chronological alterations in MDH1 immunoreactivity in the gerbil hippocampus and observed the effects of MDH1 against ischemic damage.

## Materials and methods

### Chemicals and reagents

All chemicals and reagents are specified in the text and others were purchased from Sigma (St. Louis. Mo, USA). The levels of reduced glutathione (GSH) and oxidized glutathione (GSSG), as well as the activities of MDH1, glutathione peroxidase (GPx), and glutathione reductase (GR) were determined using commercially available ELISA kits (Abcam, Cambridge, UK).

### Ethics statement

All experimental procedures were carried out in accordance with ARRIVE 2.0 guidelines^23^ and the guidelines from American Veterinary Medical Association and our institution. Experimental protocols using animals were approved by the Institutional Animal Care and Use Committee of Seoul National University (SNU-200313-2-4).

### Synthesis of Tat-MDH1 and its control protein

**Figure 1:**
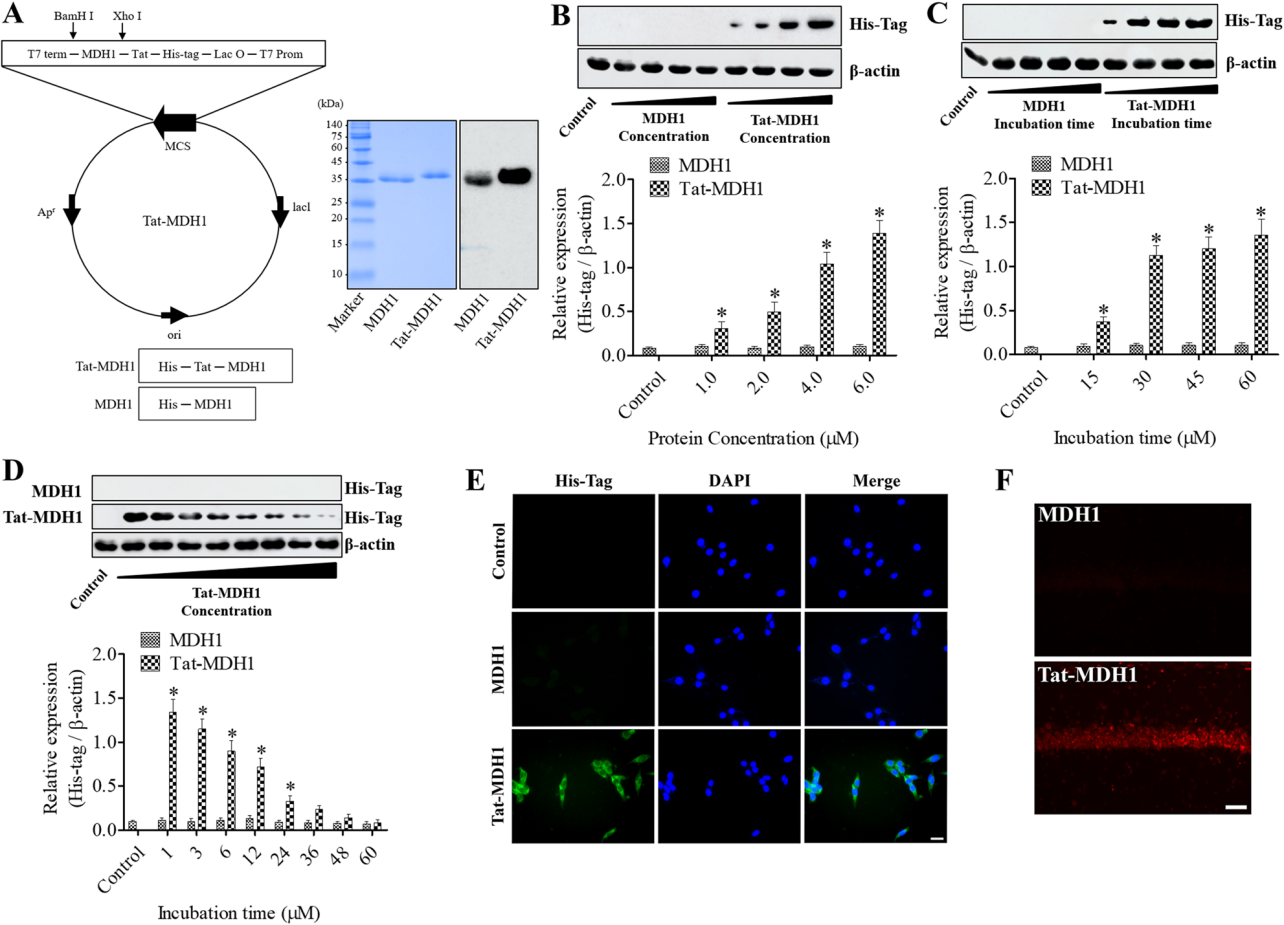
Validation of Tat-MDH1 delivery into HT22 cells and the gerbil hippocampal CA1 region. (**A**) Tat-MDH1 was designed with His-tag and MDH1 with or without the Tat expression vector. After purification, the expression of Tat-MDH1 was confirmed by Coomassie brilliant blue staining and western blotting for His-tag. (**B**) HT22 cells were incubated for 1 h after various concentration (1.0–6.0 μM) of Tat-MDH1 and MDH1 treatments. (**C**) HT22 cells were incubated for various lengths of time (15–60 min) after 4.0 μM of Tat-MDH1 and MDH1 treatments. (**D**) Tat-MDH1 (4.0 μM) and MDH1 (4.0 μM) were added to HT22 cells and harvested for various incubation time (1–60 h). Delivered proteins were detected by western blot analysis for His-tag. All assays were performed in triplicate. Data are expressed as mean + standard deviation and were analyzed using Student’s *t*-test (**B** and **C**) and one-way analysis of variance followed by Tukey’s multiple comparison post hoc test (**D**) (n = 5, *p < 0.05, significantly different from the control group). (**E** and **F**) Transduced proteins were visualized by immunochemical staining for His-tag at 1 h after 4.0 μM Tat-MDH1 treatment in HT22 cells and at 6 h after 1 mg/kg Tat-MDH1 treatment in the gerbils, respectively. Scale bar = 20 μm (**E**) and 50 μm (**F**).

To overcome the delivery problems of MDH1 into cells and the blood–brain barrier, the Tat-MDH1 fusion protein was produced using the pET15b vector (Novagen, Merck Millipore, Darmstadt, Germany) with MDH1 cDNA and a histidine-tag (His-tag), as shown in Fig. 1A. A Tat peptide expression vector was added to the Tat-MDH1 as previously described^24^. *Escherichia coli* BL21 cells (Novagen), transformed with Tat-MDH1 or MDH1 plasmids, were grown in broth media, and protein expression was induced by treatment with 0.5 mM isopropyl-β-D-thiogalactoside (Duchefa, Haarlem, the Netherlands) for 6 h at 37 °C. Transformed proteins were harvested after disruption with 5 mM imidazole, 0.5 M NaCl, and 20 mM Tris–HCl (pH 7.9) containing 6 M urea. Proteins were purified using Ni^2+^-nitrilotriacetic acid Sepharose affinity column (Qiagen, Valencia, CA, USA) and PD-10 column chromatography, and the purified protein was identified by western blotting for His-tag, as previously described^24,25^.

### Confirmation of Tat-MDH1 delivery in HT22 cells and the gerbil hippocampus

The delivery of Tat-MDH1 and MDH1 was assessed by immunostaining and western blot analysis for His-tag in HT22 cells and the gerbil hippocampus. Mouse hippocampal HT22 cells were grown in Dulbecco’s minimum essential medium (DMEM, Lonza, Walkersville, MD, USA) containing 10% fetal bovine serum, 100 μg/mL streptomycin, and 100 U/mL penicillin under 95% air and 5% CO_2_ at 37 °C. To detect the concentration- and time-dependent delivery of Tat-MDH1 and MDH1, various concentrations (0–6.0 μM) of proteins were added to HT22 cells for 1 h, and were incubated with 4.0 μM of proteins for various times. Thereafter, cells were harvested to examine His-tag expression in HT22 cells by western blotting. Intracellular degradation of proteins in HT22 cells was confirmed by western blot analysis for His-tag 60 h after 4.0 μM of protein incubation. In addition, the intracellular delivery of proteins was visualized by immunostaining for His-tag in coverslip-cultured HT22 cells 1 h after 4.0 μM of protein incubation. After harvesting, the cells were homogenized with ice-cold radioimmunoprecipitation assay buffer (Thermo Scientific). Equal concentrations of proteins were loaded onto sodium dodecyl sulfate–polyacrylamide gel electrophoresis and transferred to a polyvinylidene difluoride membrane. Thereafter, the membranes were incubated with mouse anti-His-tag (1:1,000; Abcam, Cambridge, UK) and mouse anti-β-actin (1:5,000; Cell Signaling, Danvers, MA, USA). After washing the membranes, the protein bands were visualized using chemiluminescent reagents as previously described^25,26^.

To confirm the in vivo delivery of proteins, animals (n = 5 per group) were intraperitoneally injected with 1 mg/kg Tat-MDH1 or MDH1 and anesthetized with 5% isoflurane (Baxtor, Deerfield, IL, USA). Transcardiac perfusion was performed for morphological study 6 h after ischemia/reperfusion, and both hippocampi were quickly removed for immunohistochemical staining for His-tag as previously described^25,26^. Animals were anesthetized with 5% isoflurane and transcardiac perfusion was performed via the left ventricular injection of 0.1 M phosphate-buffered saline (PBS) and 4% paraformaldehyde in 0.1 M PBS. The brain tissues were quickly removed from the skull and post-fixed in the same fixative for 12 h at 25 °C. Thereafter, the tissues were cryoprotected using 30% sucrose solution and sectioned coronally at a thickness of 30 μm using a sliding microtome with a freeze stage (HM430; Thermo Scientific, Waltham, MA, USA). Sections (between 2.0 mm and 2.7 mm caudal to the bregma) were collected based on a brain map atlas^27^ and three sections (at 150-μm intervals) were incubated with mouse anti-His-tag (1:200; Abcam) and Cy3-conjugated anti-mouse immunoglobulin G (IgG) (1:500, Jackson ImmunoResearch Inc., PA, USA) at 25 °C for 2 h.

### Protective effects of Tat-MDH1 on H_2_O_2_-induced oxidative stress in HT22 cells

Oxidative stress was induced by incubation with 200 μM hydrogen peroxide (H_2_O_2_) for 1 h and Tat-MDH1 was simultaneously treated with H_2_O_2_ in HT22 cells to assess the protective effects and optimal concentration of proteins using a water-soluble tetrazolium salt (WST-1) assay kit. In addition, surviving cells were visualized by reaction with 1 μM 5-carboxyfluorescein diacetate acetoxymethyl ester (5-CFDA AM) (Invitrogen, Carlsbad, CA, USA) with 1 μg/mL 4,6-diamidino-2-phenylindole (DAPI; Roche Applied Science, Mannheim, Germany) for 15 min at 37 °C after exposure to the optimal concentration of proteins, as previously described^25,26^.

Oxidative stress-induced DNA damage and ROS formation were determined using terminal deoxynucleotidyl transferase-mediated deoxyuridine triphosphate-biotin nick end labeling (TUNEL) and 2,7-dichlorofluorescein diacetate (DCF-DA) (Invitrogen) staining, respectively. Tat-MDH1 and 200 μM H_2_O_2_ were simultaneously treated to HT22 cells cultured on sterilized coverslips for 1 h and cells were washed with 50 mM PBS. Thereafter, the cells were treated with 4% paraformaldehyde for TUNEL staining which was performed according to the manufacturer’s instructions. To measure ROS formation, the cells were incubated with 20 μM DCF-DA with 1 μg/mL DAPI (Roche Applied Science) for 30 min, as previously described^25,26^.

The 5-CFDA AM-, DCF-DA-, and TUNEL-stained structures were captured using a fluorescence microscope (Nikon Eclipse 80i, Tokyo, Japan), and the fluorescence intensity was quantified using a Fluoroskan enzyme-linked immunosorbent assay (ELISA) plate reader (Fluoroskan Ascent, Labsystems Multiskan MCC/340, Helsinki, Finland) as previously described^25,26^.

### Changes of MDH1 immunoreactivity and activity in the gerbil hippocampus after ischemia

Three-month-old Mongolian gerbils (*Meriones unguiculatus*) were purchased from Japan SLC Inc. (Shizuoka, Japan). Ischemic surgery in the gerbils was performed as previously described^25,26^. Briefly, the animals were anesthetized with 5% isoflurane (Baxtor), and anesthesia was maintained under 2.5% isoflurane. The common carotid arteries were exposed in the neck region and occluded with aneurysm clips (Roboz Surgical Instrument Co., Inc., Gaithersburg, MD, USA) for 5 min. Body temperature during ischemic surgery was tightly regulated using a thermostat blanket connected to a rectal probe (Harvard Apparatus, Holliston, MA, USA) and infrared light beam (Daekyung Electro Co., LTD., Pocheon, South Korea). MDH1 immunohistochemical staining was performed to detect time-lapse changes in MDH1 localization in the gerbil hippocampus after ischemia as shown in Fig. 3A. In addition, MDH1 activity was determined at critical time points (6 h and 2 d) after ischemia by MDH1 immunohistochemical analysis. Five animals were used in each time point to measure the localization and activity of MDH1 in the hippocampus after ischemia. For MDH1 immunohistochemical staining, 30-μm coronal tissue sections were made after transcardiac perfusion described above and three sections (at 150-μm intervals) were sequentially incubated with rabbit anti-MDH1 (1:500, Proteintech, Rosemont, IL, USA) at 25 °C for 12 h, biotinylated goat anti-mouse IgG (1:200, Vector Laboratories, Burlingame, CA, USA) at 25 °C for 2 h, and peroxidase-conjugated streptavidin (1:200, Vector Laboratories) at 25 °C for 2 h. Thereafter, immunoreaction was developed by adding 50 mg/100 mL 3,3-diaminobenzidine tetrahydrochloride in 0.01 M PBS.

### Protective effects of Tat-MDH1 on ischemia-induced neuronal damage in gerbils

Fifteen gerbils in each group received an intraperitoneal injection of vehicle (10% glycerol), MDH-1 (1.0 mg/kg), and Tat-MDH1 (0.1, 0.3, or 1.0 mg/kg) immediately after reperfusion as shown in Fig. 4A.

The protective effects of Tat-MDH1 against ischemic damage were predicted by locomotor activity 1 d after ischemia because hyperlocomotion is a reliable characteristic of neuronal damage at an early time after ischemia before detecting morphological neuronal damage^28^. Animals (n = 5 per group) were freed in a sound-proof Plexiglas cage (25 × 20 × 12 cm), and locomotion was recorded using a digital camera system (Basler, Ahrensburg, Germany). The distance traveled and consumed time were analyzed using XT14 software (Ethovision, Wageningen, Netherlands).

The neuroprotective effects of Tat-MDH1 were confirmed morphologically 4 d after ischemia by immunohistochemical staining for neuronal nuclei (NeuN) in the hippocampus to detect surviving mature neurons as shown in Fig. 4A. Briefly, 30-μm coronal tissue sections were made after transcardiac perfusion described above and three sections (at 150-μm intervals) were sequentially incubated with mouse anti-NeuN antibody (1:1000; EMD Millipore, Temecula, CA, USA) at 25 °C for 12 h, goat anti-mouse IgG, and peroxidase-conjugated streptavidin (Vector Laboratories) at 25 °C for 2 h. Immunoreaction was developed by adding 50 mg/100 mL 3,3-diaminobenzidine tetrahydrochloride in 0.01 M PBS.

### Effects of Tat-MDH1 on ischemia-induced oxidative stress and depletion of antioxidants in gerbils

Oxidative stress and antioxidant enzyme activity were measured in the hippocampus 2 d after ischemia as shown in Fig. 5A. Briefly, animals (n = 10 per group) were re-anesthetized with 5% isoflurane, and the whole brain (n = 5 per group) was obtained to detect superoxide by visualization of dihydroethidium (DHE) fluorescence. Briefly, the tissue processing procedures were similar, but fresh whole brains were obtained without 4% paraformaldehyde fixation 2 d after ischemia. Thirty μm sections were obtained using a vibratome (Leica, Wetzlar, Germany) and incubated with 5 μM DHE solution at 25 °C for 20 min.

In addition, both fresh hippocampi (n = 5 per group) were quickly removed from the brain and lysed in ice-cold 0.1 M Tris–HCl solution to measure hydroperoxide, MDA, GSH, and GSSG levels as well as GPx and GR activities. The levels of GSH and GSSG, as well as the activities of GPx, and GR were determined using commercially available ELISA kits (Abcam), according to the manufacturer’s guidelines, as previously described^26^.

### Data quantification and analysis

For data quantification, three sections (at 150-μm intervals) were selected, and using ImageJ software version 1.53f51 (National Institutes of Health, Bethesda, MD, USA) the number of NeuN-positive neurons was counted in the stratum pyramidale which had five areas of 140 × 140 μm sampling window. The density of immunoreactive structures of MDH1, DHE fluorescence, and protein bands of His-tag and β-actin were quantified as the sum of gray scale × pixel number using ImageJ software, as previously described^25,26^. Data were normalized as percentile values versus the sham-operated group.

Data are shown as mean + standard deviation. Statistical analysis was performed using GraphPad Prism 5.01 software (GraphPad Software Inc., La Jolla, CA, USA) to compare the differences between means using Student’s *t*-test and one-way analysis of variance, followed by Tukey’s multiple comparison post hoc test.

## Results

### Confirmation of protein synthesis and their efficacy in vitro and in vivo transduction

Purified Tat-MDH1 and its control protein (MDH1) were identified based on Coomassie brilliant blue staining, and specific protein expression was assessed by western blot analysis for His-tag because a His-tag was added to the pET15b vector. The protein expression of Tat-MDH1 and MDH1 was observed at 36 kDa and 38 kDa, respectively (Fig. 1A).

Intracellular delivery of Tat-MDH1 was validated in HT22 cells using western blot analysis for His-tag, depending on various concentrations and incubation times of the proteins. Treatment with Tat-MDH1 showed a tendency to increase His-tag levels in a concentration-dependent manner, while His-tag levels did not show significant changes after MDH1 application in any concentration (Fig. 1B). Treatment with 4.0 μM Tat-MDH1 increased His-tag levels in an incubation time-dependent manner. However, MDH1 treatment did not result in any significant changes in His-tag levels depending on the incubation time (Fig. 1C).

The stability of the MDH and Tat-MDH1 protein was assessed using western blot analysis at various incubation time after 4.0 μM MDH1 or Tat-MDH1 treatment. His-tag expression was not detectable in the MDH1-treated group at any incubation time, while His-tag levels significantly increased 1 h after Tat-MDH1 treatment and decreased thereafter with incubation time after treatment. His-tag levels were significantly higher at 24 h after treatment than in the control group (Fig. 1D).

The delivery was morphologically assessed by immunocytochemical staining for His-tag 1 h after 4.0 μM MDH1 or Tat-MDH1 treatment in HT22 cells and 6 h after 1 mg/kg MDH1 or Tat-MDH1 treatment. Control and MDH1 treatment did not show any His-tag immunoreactive structures in HT22 cells, whereas in the Tat-MDH1-treated group, His-tag immunoreactivity was found in the cytoplasm of HT22 cells (Fig. 1E).

Consistent with the in vitro study, His-tag immunoreactivity was not detectable in the hippocampal CA1 region 6 h after MDH1 treatment, whereas in the Tat-MDH1-treated group His-tag immunoreactive structures were found in the hippocampal CA1 region (Fig. 1F).

### In vitro neuroprotection of Tat-MDH1 against oxidative damage in HT22 cells

**Figure 2:**
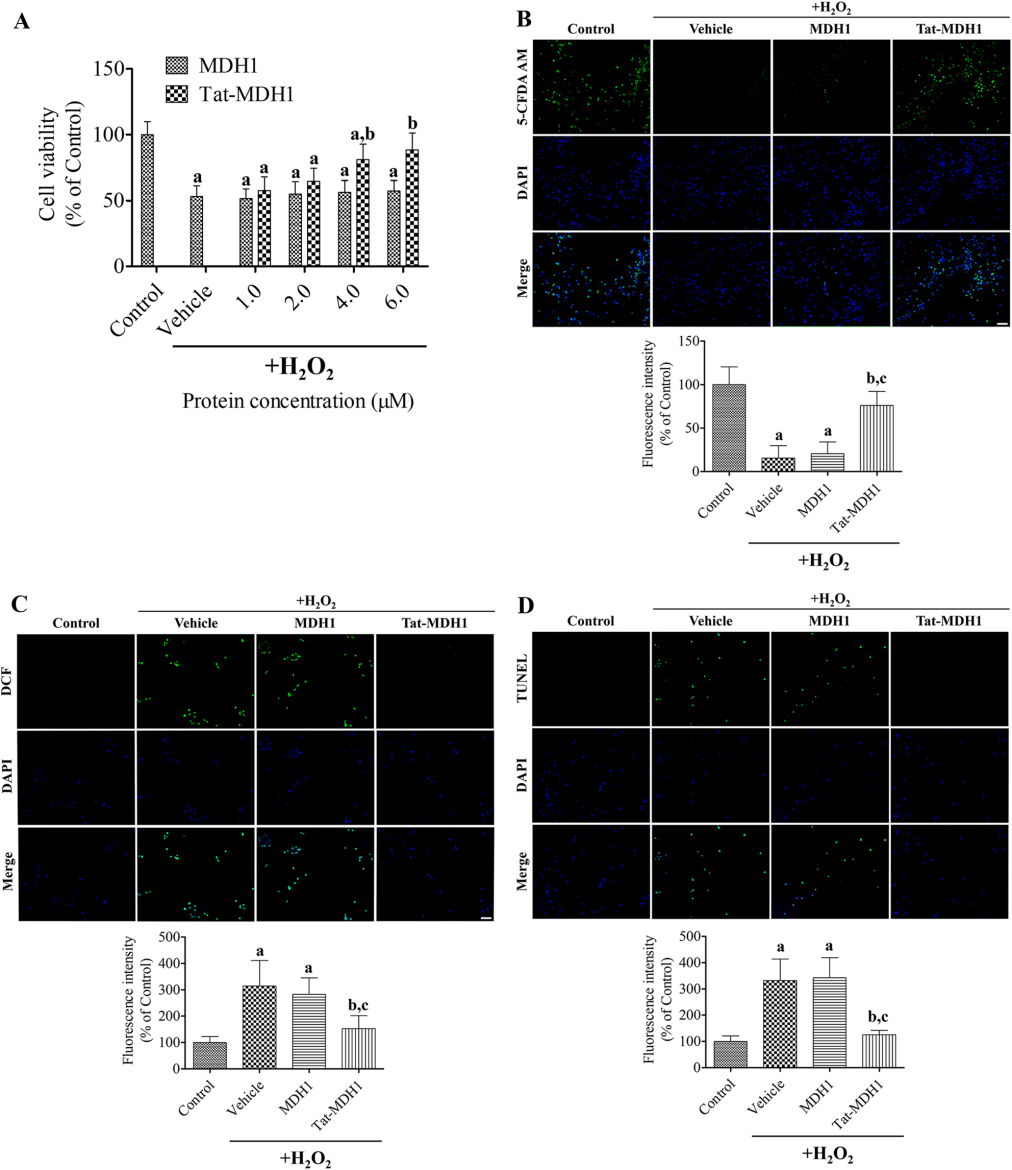
In vitro neuroprotective effects of Tat-MDH1 against 200 μM H_2_O_2_-induced oxidative stress in HT22 cells. (**A**) Concentration-dependent effects of Tat-MDH1 were observed using a water-soluble tetrazolium salt (WST-1) assay 1 h after incubation of protein and hydrogen peroxide (H_2_O_2_). (**B**–**D**) Survived cells, ROS formation, and DNA fragmentation were visualized by 5-carboxyfluorescein diacetate acetoxymethyl ester (5-CFDA AM), 2,7-dichlorofluorescein diacetate (DCF-DA), and deoxynucleotidyl transferase-mediated deoxyuridine triphosphate-biotin nick end labeling (TUNEL) staining respectively, in HT22 cells 1 h after 4 μM Tat-MDH1 and 200 μM H_2_O_2_. Scale bar = 50 μm. All assays were performed in triplicate. Fluorescence intensities were quantified and data were analyzed using one-way analysis of variance followed by Tukey’s multiple comparison post hoc test (^a^*p* < 0.05, significantly different from the control group; ^b^*p* < 0.05, significantly different from the vehicle group; ^c^*p* < 0.05, significantly different from the MDH1 group). Data are shown as mean + standard deviation.

Oxidative stress was induced by incubation with 200 μM H_2_O_2_ for 1 h, and neuroprotective effects were assessed using a WST-1 assay by simultaneous treatment with Tat-MDH1 (or MDH1) and H_2_O_2_. Vehicle treatment with H_2_O_2_ significantly decreased cell viability to 53.3% of that of the control group. Cell viability showed increases in a concentration-dependent manner after Tat-MDH1, but not MDH1, treatment. In particular, the cell viability was significantly higher in 4.0 μM (80.9% of control) and 6.0 μM (88.4% of control) Tat-MDH1-treated group than those in the vehicle-treated group (Fig. 2A).

To visualize the surviving cells, 4.0 μM MDH or Tat-MDH1 was incubated in HT22 cells with 200 μM H_2_O_2_ and 5-CFDA AM staining was performed. In the control group, 5-CFDA AM-stained cells were abundant, whereas 5-CFDA AM-stained cells were few in the vehicle-treated group. In the MDH1-treated group, a few 5-CFDA AM-stained cells were found, but there were no significant differences in the 5-CFDA AM fluorescence intensity between the vehicle- and MDH-1-treated groups. In the Tat-MDH1-treated group, many 5-CFDA AM-stained cells were detected, and the fluorescence intensity was significantly increased to 76.2% of that in the control group compared to that in the vehicle- or MDH1-treated groups (Fig. 2B).

To show the effects of Tat-MDH1 against oxidative damage in HT22 cells, HT22 cells were incubated with 200 μM H_2_O_2_ and 4.0 μM MDH or Tat-MDH1, and DCF-DA and TUNEL staining were performed, respectively. In the control group, very few DCF-stained cells were detected, while in the vehicle-treated group, DCF-stained cells were abundantly detected, and the fluorescence intensity significantly increased to 314.0% of that in the control group, respectively. In the MDH1-treated group, many DCF-stained cells were observed, and the fluorescence intensity was similar to that the vehicle-treated group. In the Tat-MDH1-treated group, a few DCF-stained cells were observed, and the fluorescence intensity was dramatically decreased to 152.5% of that in the control group, compared to those in the vehicle- or MDH1-treated groups (Fig. 2C).

Consistent with ROS formation, DNA fragmentation stained with TUNEL showed similar effects of Tat-MDH1 in HT22 cells. In the vehicle- and MDH1-treated groups, many TUNEL-stained cells were found and the fluorescence intensity showed higher levels (332.2% and 342.4%) than in the control group. In the Tat-MDH1-treated group, only a few TUNEL-stained cells were detected, and the fluorescence intensity was significantly lower (125.4% of control group) compared to those in the vehicle- or MDH1-treated groups (Fig. 2D).

### Chronological changes of MDH1 immunoreactivity and activity in CA1 region after ischemia in the gerbils

**Figure 3:**
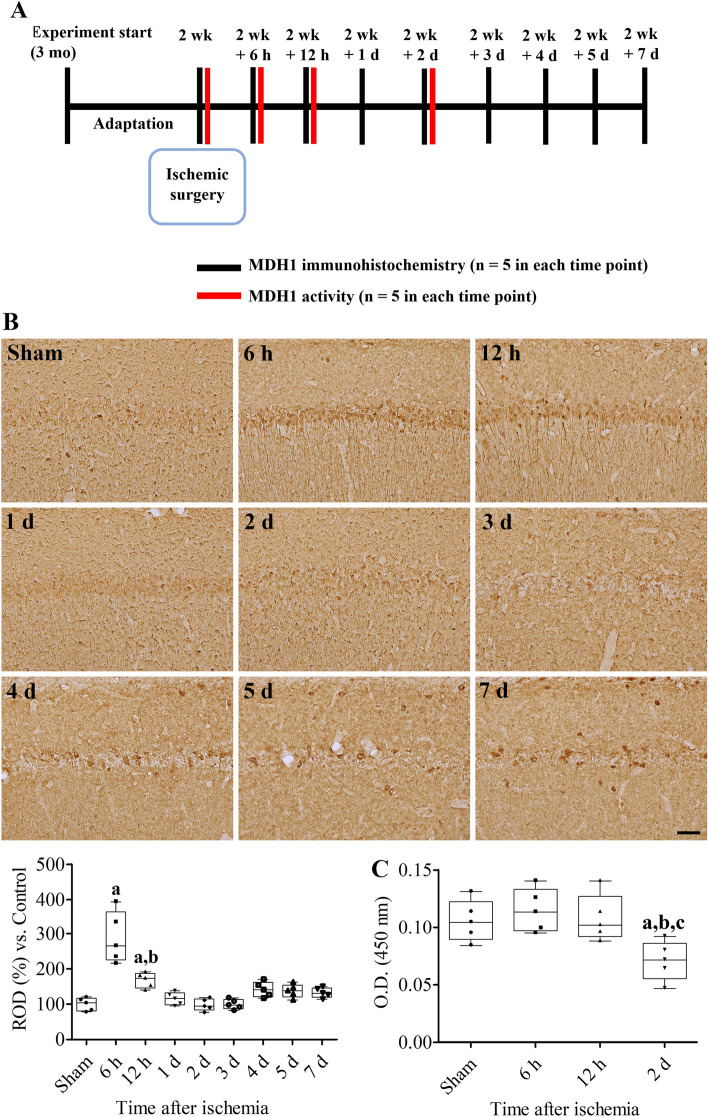
Chronological changes of MDH1 immunoreactivity and activity in the hippocampal CA1 region 5 min after ischemia in the gerbils. (**A**) Schematic drawing of the experimental study in MDH1 immunohistochemical staining and activity assay. (**B**) Spatial and temporal changes of MDH1 was assessed in the hippocampal CA1 region by immunohistochemical staining for MDH1. Scale bar = 50 μm. Optical densities are measured using ImageJ software and the relative optical density (ROD) are demonstrated as percentile values versus sham-operated group per section. (**C**) MDH1 activity was validated in the hippocampal homogenates using enzyme-linked immunosorbent assay (ELISA) kits 12 h and 2 d after ischemia. Data were analyzed using one-way analysis of variance followed by Tukey’s multiple comparison post hoc test (^a^*p* < 0.05, significantly different from the sham-operated group; ^b^*p* < 0.05, significantly different from 6 h post-ischemic group; ^c^*p* < 0.05, significantly different from the 12 h post-ischemic group). Data are shown as mean + standard deviation.

In the sham-operated group, MDH1 immunoreactivity was detected in a few cells in the CA1 region. MDH1 immunoreactive neurons were abundantly observed in the stratum pyramidale 6 h after ischemia and MDH1 immunoreactive dendrites were found in the stratum radiatum. In this group, immunoreactivity in the CA1 region was significantly increased to 289.6% of that in the control group. Thereafter, MDH1 immunoreactivity decreased with time after ischemic damage by 3 d after ischemia. From 4 to 7 d after ischemia, MDH1 immunoreactive neurons were found in the non-pyramidal cells of the CA1 region; however, their immunoreactivity was similar to those in the sham-operated group (Fig. 3B).

MDH1 activity did not show any significant changes in the hippocampal CA1 region 12 h after ischemia compared to that in the sham-operated group. However, MDH1 activity at 2 days after ischemia was significantly decreased to 67.0% of that in the sham-operated group compared to that at 6 or 12 h after ischemia (Fig. 3C).

### In vivo neuroprotection of Tat-MDH1 against ischemic damage in the gerbils

**Figure 4:**
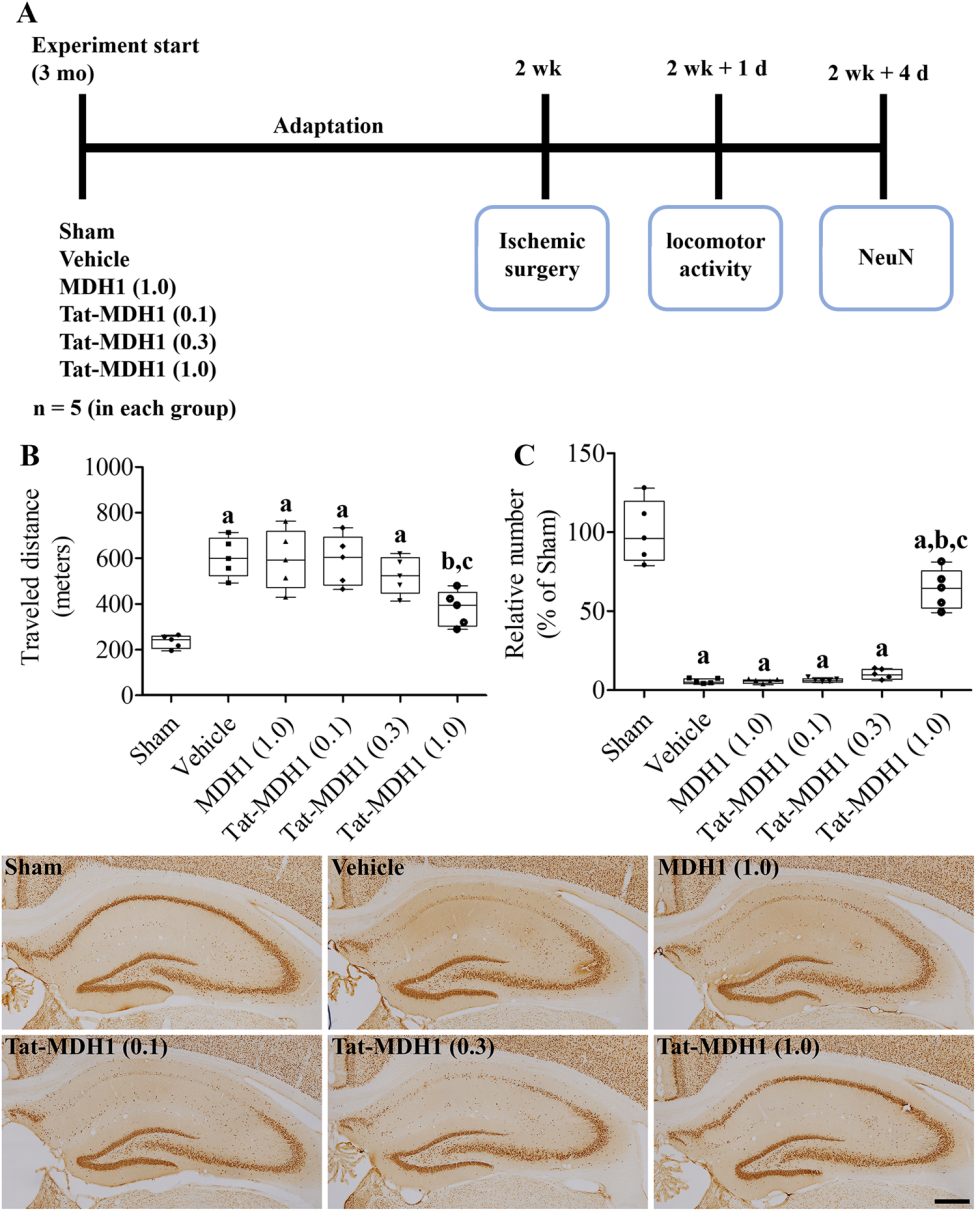
In vivo neuroprotective effects of Tat-MDH1 against 5 min of ischemic damage in gerbils. (**A**) Schematic drawing of the motor activity test and immunohistochemical staining for NeuN. (**B**) Locomotor activity was recorded and distance traveled was traced 1 d after ischemia in the sham-operated (sham), Tat peptide (vehicle)-treated, 1.0 mg/kg MDH1-treated [MDH1 (1.0)], and 0.1 mg/kg MDH1-treated [MDH1 (0.1)], 0.3 mg/kg MDH1-treated [MDH1 (0.3)], and 1.0 mg/kg Tat-MDH1-treated [Tat-MDH1 (1.0)] groups. (**C**) Surviving neurons 4 d after ischemia were visualized by immunohistochemical staining for NeuN in the hippocampus. Scale bar = 400 μm. The number of NeuN-immunoreactive neurons in CA1 region was counted, and the percentile number versus sham-operated group was demonstrated. Data were analyzed using one-way analysis of variance followed by Tukey’s multiple comparison post hoc test (^a^*p* < 0.05, significantly different from the sham-operated group; ^b^*p* < 0.05, significantly different from the vehicle-treated group; ^c^*p* < 0.05, significantly different from the MDH1-treated group). Data are shown as mean + standard deviation.

Spontaneous motor activity was monitored 1 d after ischemia because hyperlocomotion is one of the characteristics of hippocampal damage after ischemia in the gerbils^29^. In the vehicle- and MDH1-treated groups, the gerbils showed higher motor activity, and distance traveled dramatically increased to 255.4% and 253.1% of those in the sham-operated group, respectively. The distance traveled decreased in a concentration-dependent manner after Tat-MDH1 treatment, however, a significant reduction in motor activity was only found after 1.0 mg/kg Tat-MDH1 treatment. The distance traveled was 162.1% of that in the sham-operated group (Fig. 4B).

Surviving neurons were visualized using immunohistochemical staining for NeuN in the hippocampus 4 d after ischemia. In the sham-operated group, NeuN-immunoreactive neurons were abundant in the stratum pyramidale of the CA1-3 region and the granule cell layer of the dentate gyrus. In the vehicle- and MDH1-treated groups, a few NeuN-immunoreactive neurons were detected in the stratum pyramidale of the CA1 region, whereas they were abundant in other regions. In the vehicle- and MDH1-treated groups, the number of NeuN neurons in the CA1 region was 5.5% and 5.4% of those in the sham-operated group, respectively. More NeuN-immunoreactive neurons were detected, depending on the concentration of Tat-MDH1. In the 1.0 mg/kg Tat-MDH1-treated group, NeuN-immunoreactive neurons were abundantly found in the CA1 region, and the number was significantly higher (64.0% of that in the sham-operated group) than that in the vehicle- or MDH1-treated groups (Fig. 4C).

### Effects of Tat-MDH1 on ischemia-induced oxidative stress in the gerbils

**Figure 5:**
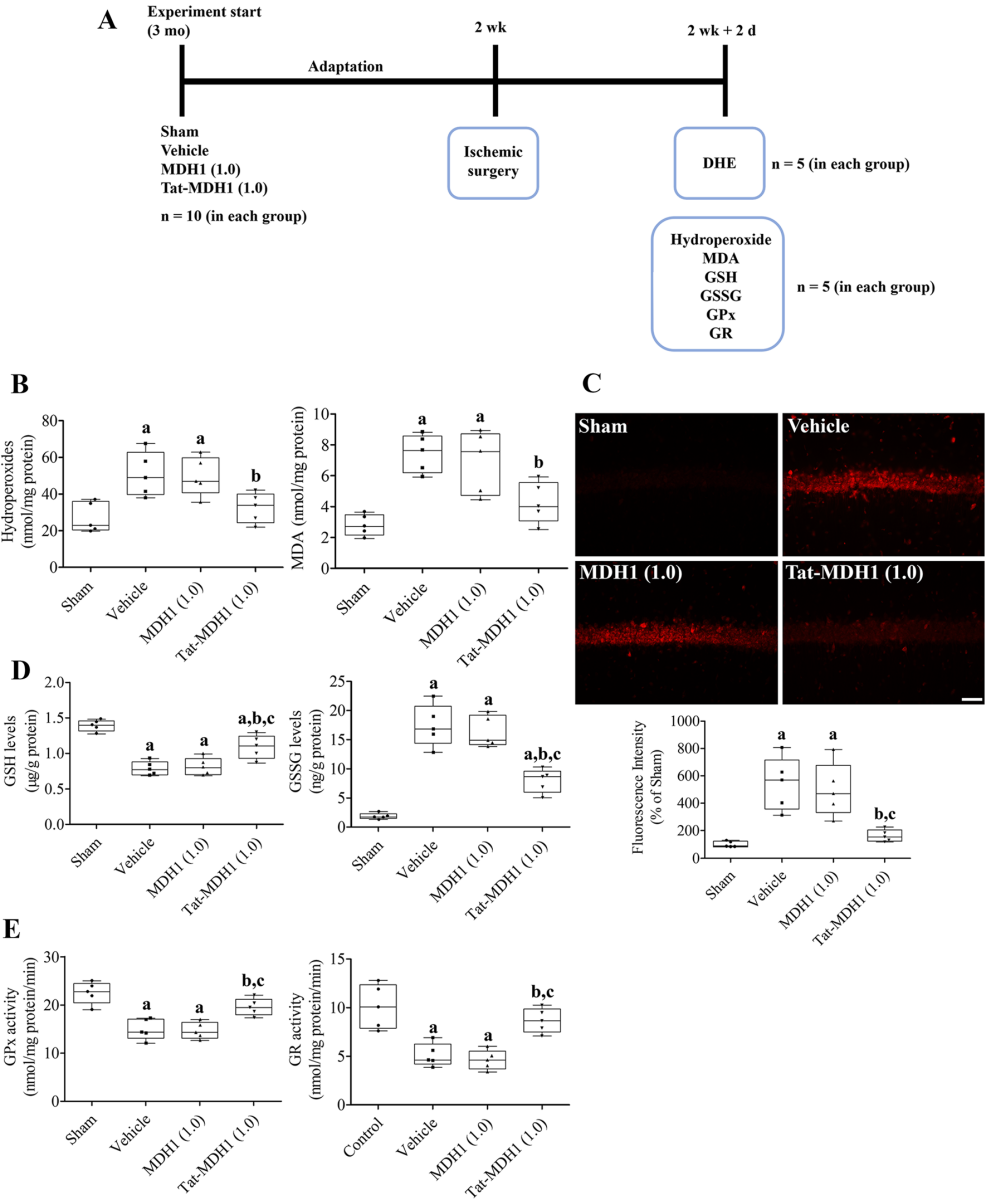
In vivo effects of Tat-MDH1 on oxidative stress and GSH system in hippocampus 2 days after ischemia. (**A**) Schematic drawing of the experimental protocol used for measuring oxidative markers and reduced glutathione (GSH) systems. (**B**) Oxidative stress was assessed by measurements of hydroperoxides and malondialdehyde (MDA) in the hippocampus. (**C**) Oxidative damage is visualized by dihydroethidium (DHE) fluorescence in the hippocampus. (**D** and **E**) Levels of reduced GSH and oxidized glutathione (GSSG) as well as glutathione peroxidase (GPx) and glutathione reductase (GR) activities were determined in the hippocampus 2 d after ischemia. Data were analyzed using one-way analysis of variance followed by Tukey’s multiple comparison post hoc test (^a^*p* < 0.05, significantly different from the sham-operated group; ^b^*p* < 0.05, significantly different from the vehicle-treated group; ^c^*p* < 0.05, significantly different from the 1 mg/kg MDH1-treated group [MDH1 (1.0)]). Data are shown as mean + standard deviation.

Ischemia-induced oxidative stress was determined by measuring hydroperoxide and MDA levels in the hippocampus using ELISA assay kits. In addition, superoxide was visualized using DHE staining of unfixed hippocampal sections. In the vehicle- and 1.0 mg/kg MDH1-treated groups, hydroperoxide levels were significantly increased to 187.0% and 182.8% of those in the sham-operated group, respectively. However, in the 1.0 mg/kg Tat-MDH1-treated group, hydroperoxide levels significantly decreased to 119.8% of those in the sham-operated group compared to those in the vehicle-treated group. Similarly, MDA levels in vehicle- and 1.0 mg/kg MDH1-treated groups were significantly increased to 265.7% and 246.0% of those in the control group, respectively. In the 1.0 mg/kg Tat-MDH1-treated group, MDA levels were significantly decreased to 152.1% of those in the control group compared to those in the vehicle-treated group (Fig. 5B).

In the sham-operated group, DHE-stained cells were very few and the fluorescence intensity was very weak in the hippocampal CA1 region. In the vehicle- and 1.0 mg/kg MDH1-treated groups, strong DHE-stained structures were found in the stratum pyramidale of the CA1 region; the fluorescence intensities were significantly increased to 543.4% and 497.7% of those in the sham-operated group, respectively. In the 1.0 mg/kg Tat-MDH1-treated group, weak to moderate DHE-stained cells were found in the stratum pyramidale of the CA1 region, and the fluorescence intensity was significantly decreased to 161.8% of that in the control group compared to those in the vehicle- or 1.0 mg/kg MDH1-treated groups (Fig. 5C).

GSH has the potential to scavenge ROS and is converted to its oxidized form (GSSG). In the sham-operated group, the GSH and GSSG levels in the hippocampus were 1.391 μg/g and 1.862 ng/g, respectively. In the vehicle-treated group, GSH levels were significantly decreased to 56.5% of those in the sham-operated group, whereas GSSG levels were dramatically increased to 935.1% of those in the sham-operated group. In the 1.0 mg/kg MDH1-treated group, GSH and GSSG levels were similar to those in the vehicle-treated group. However, in the 1.0 mg/kg Tat-MDH1-treated group, GSH and GSSG levels were significantly higher and lower (78.4% and 427.6% of those in the sham-operated group) than those in the vehicle- or MDH1-treated groups, respectively (Fig. 5D).

GPx detoxifies hydroperoxide and oxidizes GSH into GSSG. GSSG is reduced by GR, which catalyzes disulfide into sulfhydryl and is believed to be a critical enzyme that maintains redox homeostasis in cells. In the sham-operated group, GPx and GR activities were 22.5 and 10.1 nmol/mg protein·min, respectively. In the vehicle-treated group, GPx and GR activities significantly decreased to 66.4% and 50.5% of those in the sham-operated group, respectively. In the 1 mg/kg MDH1-treated group, they were similar to those in the vehicle-treated group, whereas in the 1 mg/kg Tat-MDH1-treated group, GPx and GR activities showed significant amelioration to 86.9% and 85.7% of those in the sham-operated group, respectively, compared to those in vehicle- or MDH1-treated groups (Fig. 5E).

## Discussion

MDH is the final acceptor in the TCA cycle and its expression of MDH1 is highly associated with metabolic demands in a tissue-specific manner^12,30,31^. ROS induced by ischemia/reperfusion overwhelms the antioxidant defense mechanism in cells^32,33^ and affects the structure and function of proteins susceptible to oxidative stress. Recently, Guo et al. demonstrated oxidative modification of MDH1 at Cys137 in the brains of aging mice^21^. In the present study, we investigated the effect of MDH1 against oxidative damage in HT22 cells and ischemic damage in the gerbils, because MDH1 may be highly susceptible to oxidative stress and may be modified after ischemia.

We prepared a Tat-MDH1 fusion protein to deliver it to the intracellular space in HT22 cells or to the brain in the gerbils because MDH1 has a high molecular weight to penetrate cells or the blood–brain barrier. The Tat peptide (11-mer peptide, TGRJJRRQRRR) originates from the human immunodeficiency virus and enables translocation of the protein into cells or across the blood–brain barrier^34^. In the present study, we observed the concentration- and time-dependent delivery of the Tat-MDH1 into HT22 cells by measuring His-tag, which was inserted into the pET15b vector. In contrast, the control MDH1 protein did not show any significant effects, depending on its concentration or incubation time. We observed degradation of the delivered 4.0 μM Tat-MDH1 and morphological evidence of protein delivery. In addition, we confirmed the delivery of 1 mg/kg Tat-MDH1 into the gerbil hippocampus 6 h after ischemia. These results are consistent with previous studies showing that Tat-cargo were efficiently and effectively translocated into HT22 cells and the gerbil hippocampus^24,25,35,36^. In this study, we observed that treatment with H_2_O_2_ significantly decreased cell viability, and simultaneous treatment with H_2_O_2_ and Tat-MDH1 ameliorated the reduction in cell viability in a concentration-dependent manner, whereas surviving cells were significantly increased after treatment with 4 μM Tat-MDH1 based on 5-CFDA-AM staining. In addition, treatment with Tat-MDH1, but not with MDH1, significantly ameliorated H_2_O_2_-induced DNA fragmentation and ROS production in HT22 cells. This result is consistent with that of a previous study showing that MDH1 significantly reduced ROS production in hemin-stimulated HT22 cells^37^.

Next, we investigated the chronological changes in MDH1 immunoreactivity in the hippocampal CA1 region because several studies have demonstrated conflicting evidences regarding expression of MDH1 levels or activities after oxidative stresses such as hypoxia and aging^15,21,38^. In the present study, we observed that MDH1 immunoreactivity transiently and significantly increased in the CA1 region 6–12 h after ischemia. In contrast, MDH1 activity was maintained 6 h after ischemia, however significantly decreased 2 d after ischemia. This result suggests that MDH1 transiently increased 6 h after ischemia; however, MDH1 function may decrease 2 d after ischemia. This result is in agreement with a previous study showing that MDH1 levels were decreased in the hippocampus after 24 h of hypoxia^15^. In the aged brain, MDH1 levels increased in an age-dependent manner, whereas its activity was lower in the tissue of older brains than in the young or middle-aged brains, presumably due to oxidative damage during aging process^21^. In addition, Guo et al. hypothesized that MDH1 expression increases owing to a compensatory response in the trioxidation of MDH1^21^. Previously, we observed an increase in thioredoxin 2, a major redox enzyme of Cys residues in antioxidants, 1 day after ischemia/reperfusion^39^.

To elucidate the correlation between the reduction in MDH1 activity and neuronal death after ischemia, we treated Tat-MDH1 into the gerbils immediately after ischemia/reperfusion. Tat-MDH1 treatment alleviated ischemia-induced increases in locomotor activity 1 d after ischemia and neuronal damage 4 d after ischemia in a concentration-dependent manner. In addition, treatment with Tat-MDH1 significantly ameliorated ischemia-induced hydroperoxide levels, MDA levels, and DHE fluorescence in the hippocampus 2 d after ischemia. This result was supported by a previous study showing that MDH1 overexpression significantly reduced MDA levels induced by intracranial hemorrhage^37^. In the present study, we focused on the GSH status after ischemia because it protects neurons from oxidative or ischemic damage as a major intracellular antioxidant^40,41^. GSH oxidized into GSSG reacted with GPx, which detoxified hydroperoxide, and GSH was reduced to its naïve form by reducing enzymes such as GR. Transient forebrain ischemia significantly decreased GSH levels, as well as GPx and GR activities in the hippocampus. However, treatment with Tat-MDH1 significantly ameliorated the reduction in GSH levels, as well as GPx and GR activities. This result suggests that the increase in GSH status by Tat-MDH1 may reduce the oxidation of Cys residues of antioxidants to sulfenic acid, sulfinic acid, or sulfonic acid. However, the status of Cys residue oxidation in the hippocampus after Tat-MDH1 treatment remained to be elucidated.

Incidence and risk of stroke increases with age, whereas MDH1 is susceptible to alterations during aging^21^. There have been reports that MDH1 could accelerate cancer cell proliferation^42^ and survival^43^. The limitation of this study design is that the presence of a tumor must be confirmed to administer Tat-MDH1 to ischemic patients.

In conclusion, Tat-MDH1 has a neuroprotective potential against H_2_O_2_-induced oxidative stress in HT22 cells and ischemia-induced ischemic damage in the gerbils. This effect is associated with a reduction in oxidative stress and maintenance of the GSH-related redox system in the hippocampus.

## Supplementary Information

Supplementary Information.

## Data Availability

The datasets used and/or analysed during the current study available from the corresponding author on reasonable request.
