# Supplementary figures and images for: Tat-malate dehydrogenase fusion protein protects neurons from oxidative and ischemic damage by reduction of reactive oxygen species and modulation of glutathione redox system
Source: Sci Rep. 2023 Apr 6;13:5653. doi: 10.1038/s41598-023-32812-0 (PMC10079925; doi:10.1038/s41598-023-32812-0)

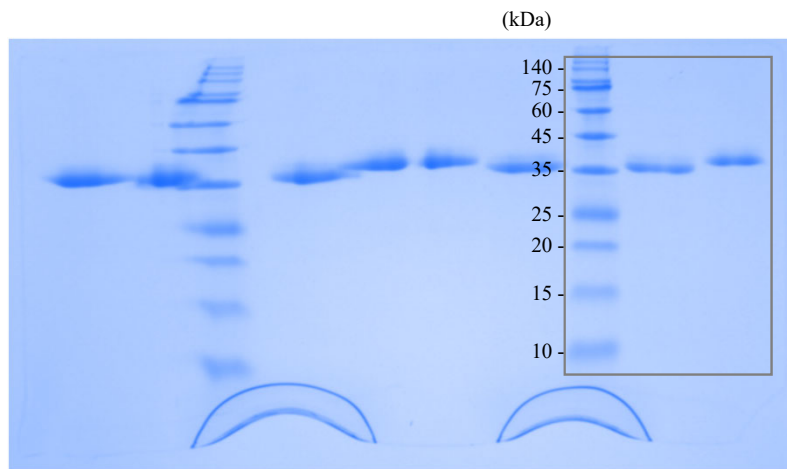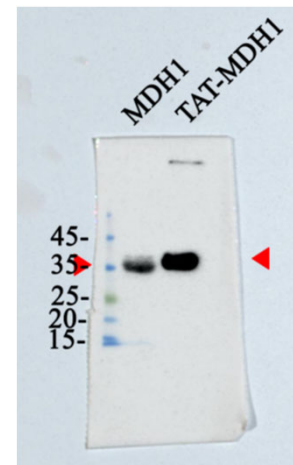

Figure 1A

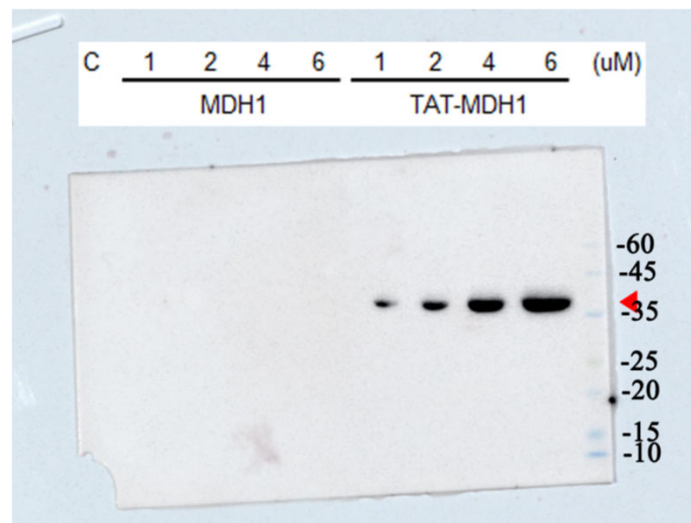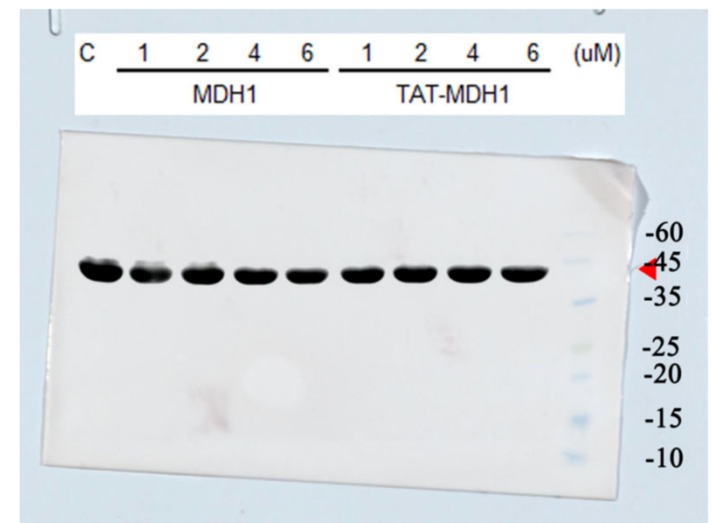

Figure 1B

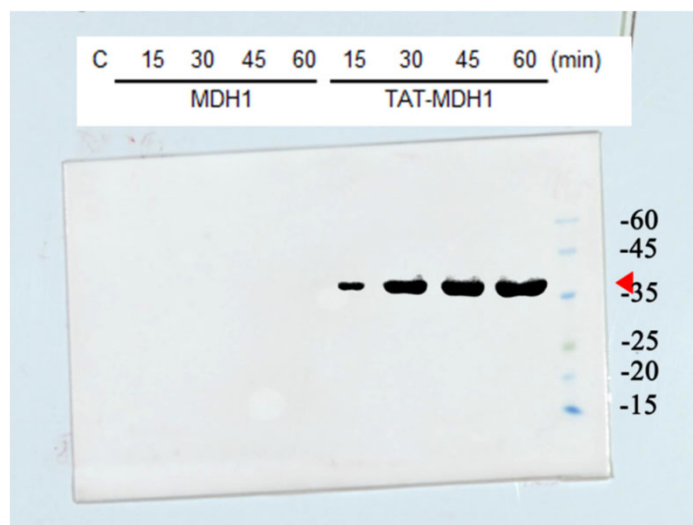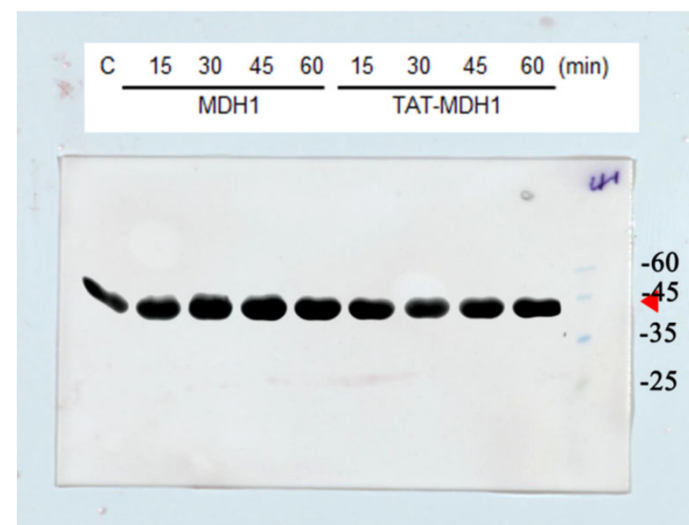

Figure 1C

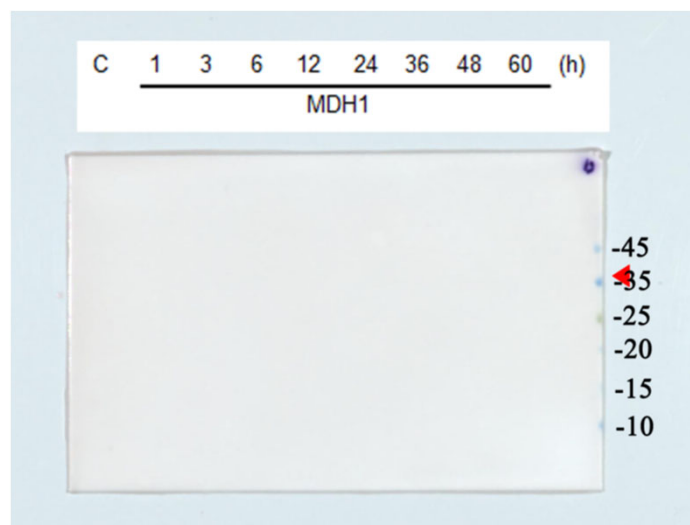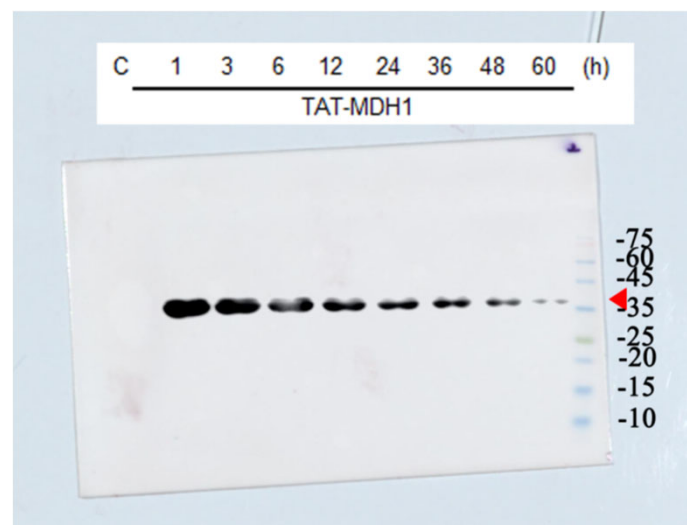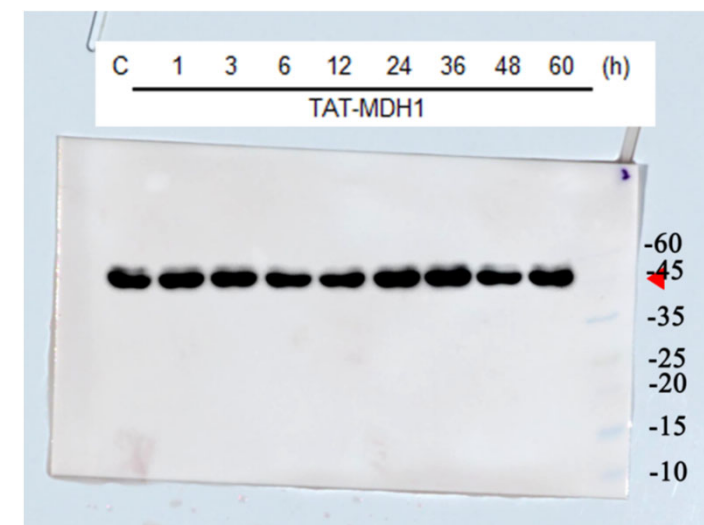

Figure 1D

Supplement: Supplementary file 1 — Supplementary Information. [file 41598_2023_32812_MOESM1_ESM.pdf]
